# Robot-assisted laparoscopic simultaneous bilateral partial nephrectomy for bilateral renal carcinoma: a case report

**DOI:** 10.3389/fmed.2026.1722040

**Published:** 2026-01-22

**Authors:** Zhenyu Cui, Ce Qin, Yong Suo, Shichao Song, Hongmei Li, Tao Ma

**Affiliations:** Department of Urology, Affiliated Hospital of Hebei University, Baoding, Hebei, China

**Keywords:** bilateral renal carcinoma, partial nephrectomy, robot-assisted, robotic surgery, simultaneous surgery

## Abstract

**Background:**

Renal carcinoma is one of the most common malignant tumors of the urinary system, with more than 400,000 new cases diagnosed worldwide each year. More than 90% of cases involve unilateral tumors, whereas sporadic synchronous bilateral renal carcinoma (sBRC) accounts for only 1–5% of patients.

**Case description:**

A 38-year-old male patient was admitted to Affiliated Hospital of Hebei University with a 5-day history of bilateral renal masses detected during a routine health examination. The patient with sporadic sBRC using robot-assisted laparoscopic simultaneous bilateral partial nephrectomy via a retroperitoneal approach. The procedure achieved favorable clinical outcomes.

**Conclusion:**

With the advancement of robotic surgery and improvements in surgical expertise, simultaneous bilateral partial nephrectomy has emerged as a viable alternative. Surgical decision-making must consider tumor stage and R. E. N. A. L. nephrometry score.

## Introduction

Renal carcinoma is one of the most common malignant tumors of the urinary system, with more than 400,000 new cases diagnosed worldwide each year (1, 2). More than 90% of cases involve unilateral tumors, whereas sporadic synchronous bilateral renal carcinoma (sBRC) accounts for only 1–5% of patients (3). Currently, there is no standardized surgical strategy for sporadic sBRC. Most institutions recommend a staged surgical approach to minimize the impact on renal function (4). Robot-assisted laparoscopic partial nephrectomy (RLPN) is increasingly being recognized as the standard surgical technique for nephron-sparing surgery. Compared with conventional laparoscopy, RLPN is associated with lower rates of conversion to open or radical surgery, shorter renal warm ischemia times, and less impairment of glomerular filtration rate. These advantages make simultaneous bilateral partial nephrectomy a feasible treatment option for sBRC (5, 6). The Department of Urology at the Affiliated Hospital of Hebei University recently treated a patient with sporadic sBRC using robot-assisted laparoscopic simultaneous bilateral partial nephrectomy via a retroperitoneal approach. The procedure achieved favorable clinical outcomes. This case is reported below.

## Case report

**Figure 1:**
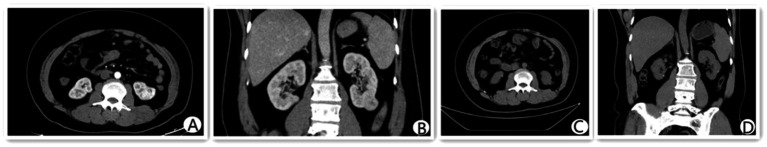
Preoperative and 1-year postoperative CT images of a patient with sBRC. Preoperative axial image **(A)** and coronal image **(B)** Axial image **(C)** and coronal image **(D)** at 1 year postoperative.

A 38-year-old male patient was admitted to our hospital with a 5-day history of bilateral renal masses detected during a routine health examination. The patient had a body mass index (BMI) of 27.4 kg/m^2^. He had a 3-year history of hypertension, with well-controlled blood pressure on oral medication. On admission, his blood pressure was 140/90 mmHg, and serum creatinine was 95 μmol/L and estimated glomerular filtration rate (eGFR) was 88.6 mL/min/1.73m^2^. Physical examination of the heart, lungs, and abdomen revealed no abnormalities. Non-contrast and contrast-enhanced computed tomography (CT) scans demonstrated nodular soft tissue-density lesions protruding beyond the renal contour on both kidneys. The larger lesion was found in the left kidney, measuring approximately 3.5 × 2.8 cm, while the right renal mass measured about 1.5 cm in diameter. Both lesions had well-defined margins and exhibited rapid enhancement and washout (Figures 1A,B). These findings strongly suggested clear cell renal cell carcinoma (ccRCC). He had no family history of renal cell carcinoma and tested negative for von Hippel–Lindau (VHL) gene mutations. The preoperative diagnosis was bilateral renal masses, both staged as T1aN0M0, with R. E. N. A. L. nephrometry scores of 6X (left) and 6P (right).

**Figure 2:**
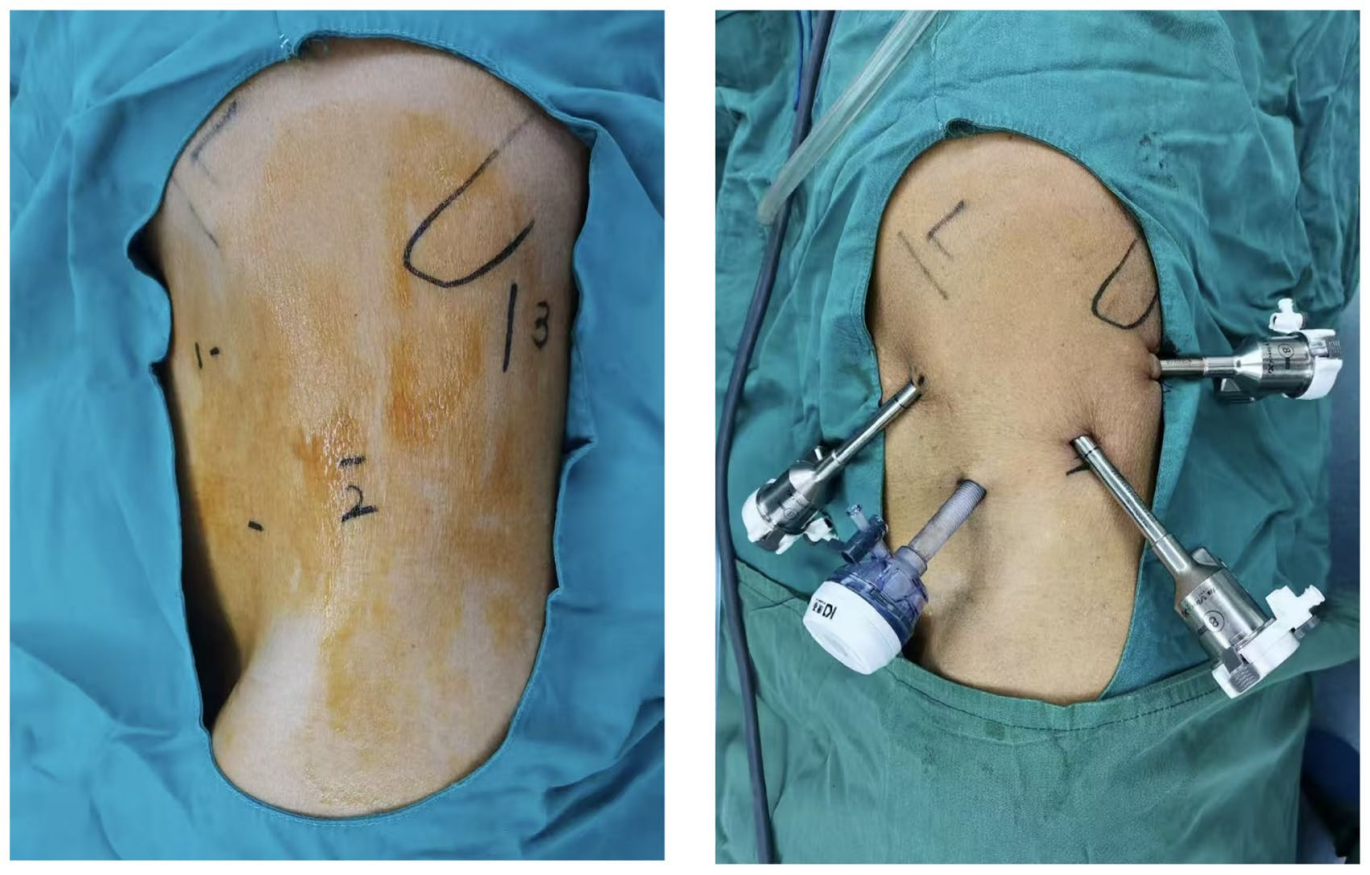
The positions of the trocar placements (essentially identical on both sides).

**Figure 3:**
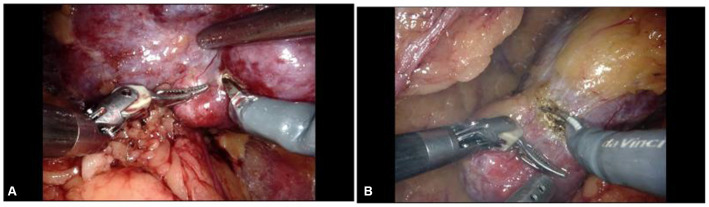
Intraoperative views of robot-assisted laparoscopic simultaneous bilateral partial nephrectomy. **(A)** Right renal tumor; **(B)** left renal tumor.

Given the patient’s good general condition, the relatively small tumor size, and his strong preference for simultaneous surgery, robot-assisted laparoscopic bilateral partial nephrectomy was performed. Under general anesthesia, the patient was first placed in the right lateral decubitus position. The retroperitoneal space and robotic access ports were established using standard techniques (Figure 2). The left renal artery was mobilized, and the perirenal fat was dissected along the renal capsule. A round, approximately 2.5 cm mass was identified at the lower pole of the left kidney. The left renal artery was clamped with a vascular bulldog clamp, and the tumor, along with a margin of adjacent renal parenchyma, was excised. The resection bed was closed with a running barbed suture. After removal of the vascular clamp, the kidney regained a satisfactory color. A drain was placed, and the incision was closed. The patient was then repositioned to the left lateral decubitus position, and the right renal tumor was completely excised in the same manner (Figure 3).

The procedure was completed successfully. The duration of operation was 175 min; intraoperative blood loss was 30 mL for the left-sided procedure and 25 mL for the right. The warm ischemia time was approximately 23 min for the left kidney and 20 min for the right. The total clamping time was 22 min for the right side and 25 min for the left side. The early unclamping technique was employed intraoperatively. The patient recovered uneventfully without perioperative complications.(Clavien–Dindo 0) Postoperative histopathology revealed bilateral renal cell carcinoma, most consistent with ccRCC. Pathological T stage was pT1a, without lymphovascular invasion, and the tumor was situated 5 mm from the surgical margin. According to the World Health Organization/International Society of Urological Pathology grading system, both tumors were graded as 1–2. The maximum tumor diameters were 2.5 cm (left) and 1.5 cm (right). Neither tumor invaded the renal capsule, and surgical margins were negative for malignancy. On postoperative day 1, the patient’s serum creatinine was 96 μmol/L (eGFR:87.3 mL/min/1.73m^2^). The drainage tube was removed on the third postoperative day, while the urinary catheter was removed on the second postoperative day. Serum creatinine measured at discharge was 95.7 μmol/L (eGFR:87.4 mL/min/1.73m^2^), and it remained stable at 95.5 μmol/L (eGFR:87.7 mL/min/1.73m^2^) one month after the operation. He was followed up regularly in the outpatient clinic. At the 1-year follow-up, CT imaging demonstrated normal renal morphology without evidence of recurrence or distant metastasis (Figures 1C,D).

## Discussion

Surgery remains the mainstay of treatment for sporadic sBRC, as it plays a critical role in prolonging survival, preserving renal function, and improving quality of life (4). Sporadic synchronous bilateral renal cell carcinoma exhibits staging-dependent prognosis. Qi et al. (5) reported 5-year OS of 92.3% for T1 tumors versus 31.8% for T4/M1 cases. Similarly, Di Maida et al. (6) observed 94.2% 5-year OS in T1a disease, declining to 52.8% for T3/T4 stages. The choice between simultaneous and staged surgery does not follow absolute indications and should be individualized based on the patient’s general condition, comorbidities, and personal preference. Some studies have suggested that simultaneous bilateral surgery may place greater stress on renal function, with a subset of patients requiring renal replacement therapy postoperatively (7). Consequently, many surgeons prefer staged procedures.

Surgical options for sporadic sBRC include radical nephrectomy and partial nephrectomy. The latter is considered safe and effective, achieving both satisfactory oncological control and renal function preservation (5). Regarding surgical approach, both radical and partial nephrectomy can be performed via open surgery, conventional laparoscopy, or robot-assisted laparoscopy. In recent years, RLPN has become increasingly prevalent in nephron-sparing surgery. Compared with open surgery, RLPN is associated with a significantly lower rate of perioperative complications. Compared with conventional laparoscopy, it allows shorter warm ischemia time and reduces the risk of postoperative renal function decline (8). Moreover, robotic systems provide enhanced instrument stability, superior anatomical precision, and a magnified three-dimensional surgical field, which collectively reduce the risk of positive surgical margins and potentially improve oncological outcomes (6). In addition, the clinical applications of RLPN continue to expand. Favorable surgical outcomes have been reported in challenging scenarios, including entirely endophytic tumors, large-volume tumors (cT2–T3), solitary kidneys, recurrent tumors, and hilar renal masses (9). Gallo et al. (10) further investigated the feasibility and clinical outcomes of simultaneous robotic partial nephrectomy (SBRPN) in patients with bilateral renal tumors. Their study highlighted that performing both resections during a single anesthesia and single surgical session avoids the physiological and psychological burden associated with staged operations. Leveraging the precision and stability of the robotic platform, SBRPN enables complete oncological resection while maximizing nephron preservation. The existing evidence supports that synchronous bilateral robotic partial nephrectomy (SBRPN) represents a safe, effective, and nephron-sparing treatment strategy, provided that patients are appropriately selected based on specific clinical and anatomical criteria. Ideal candidates should demonstrate well-preserved preoperative renal function (e.g., eGFR ≥ 80 mL/min/1.73 m^2^), absence of significant comorbidities, and physiological tolerance to extended anesthesia. Tumor characteristics should be limited to T1 stage (≤7 cm), low-to-intermediate anatomical complexity, and no evidence of distant metastasis. Additionally, a prerequisite is the uneventful completion of the first-side procedure with minimal intraoperative blood loss (11, 12). This approach provides an important minimally invasive alternative for the management of bilateral renal tumors. Accordingly, SBRPN holds promise as a potential standard of care for patients with sBRC.

This case employed a retroperitoneal approach, which eliminates the need for retraction of abdominal organs and minimizes interference with the intestines, leading to faster postoperative recovery of gastrointestinal function. Moreover, this approach provides direct access to the renal fascia, thereby shortening the surgical pathway. Compared with the transperitoneal approach, the operative time in this case was 175 min, which is notably shorter than the reported 240–260 min for some simultaneous bilateral surgeries performed via the transperitoneal approach. The bilateral warm ischemia times were 23 min and 20 min, respectively, lower than the typical 25–30 min associated with the transperitoneal approach. No blood transfusion or serious complications occurred, and both the transfusion rate and overall complication rate were better than those reported in studies on the transperitoneal approach. While reducing the risk of injury to abdominal organs, the retroperitoneal approach further optimizes key surgical indicators through a shorter operative pathway, making it particularly suitable for patients with bilateral lesions located on the dorsal or middle aspects of the kidneys (13).

Whether patients with bilateral renal carcinoma have worse prognoses than those with unilateral disease remains controversial. Several studies have reported comparable outcomes between sporadic sBRC and unilateral renal carcinoma (7, 14). However, other studies have demonstrated that patients with sporadic sBRC have significantly lower 5-year relapse-free survival and overall survival compared with unilateral cases (15). Prognostic factors for sporadic sBRC include patient age, tumor size and stage, pathological subtype, treatment modality, and surgical margin status (15).

## Conclusion

In summary, sporadic sBRC is rare in clinical practice, and surgery remains the cornerstone of treatment. Traditionally, staged procedures have been preferred in order to preserve renal function. However, with the advancement of robotic surgery and improvements in surgical expertise, simultaneous bilateral partial nephrectomy has emerged as a viable alternative. Surgical decision-making must consider tumor stage and R. E. N. A. L. nephrometry score. Given the ongoing controversy regarding prognosis, patients with sBRC require closer postoperative follow-up.

## Data Availability

The raw data supporting the conclusions of this article will be made available by the authors, without undue reservation.

## References

[1] Bahadoram S Davoodi M Hassanzadeh S Bahadoram M Barahman M Mafakher L. Renal cell carcinoma: an overview of the epidemiology, diagnosis, and treatment. G Ital Nefrol. (2022) 39:2022-vol3. 35819037

[2] Chandramohan D Garapati HN Nangia U Simhadri PK Lapsiwala B Jena NK . Diagnostic accuracy of deep learning in detection and prognostication of renal cell carcinoma: a systematic review and meta-analysis. Front Med. (2024) 11:1447057. doi: 10.3389/fmed.2024.1447057, 39301494 PMC11412207

[3] Fuentes AE Ramírez RA Vera GP Del Valle FA Bruzzone AE Rojas FA . Synchronous giant bilateral renal tumors as initial presentation of Von Hippel-Lindau disease: sequential surgical management and transition to renal replacement therapy. Urol Case Rep. (2025) 62:103150. doi: 10.1016/j.eucr.2025.103150, 40837127 PMC12362395

[4] Pirzada FM Kumar S Agarwal K Nayak B. Von Hippel-Lindau syndrome with bilateral renal and an interaortocaval mass. BMJ Case Rep. (2025) 18:e263804. doi: 10.1136/bcr-2024-263804, 40055011

[5] Qi N Li T Ning X Peng X Cai L Gong K. Clinicopathologic features and prognosis of sporadic bilateral renal cell carcinoma: a series of 148 cases. Clin Genitourin Cancer. (2017) 15:618–24. doi: 10.1016/j.clgc.2017.03.008, 28648756

[6] Di Maida F Grosso AA Sforza S Mari A Lambertini L Nardoni S . Surgical management of synchronous, bilateral renal masses: a 1-decade referral center experience. Eur Urol Focus. (2022) 8:1309–17. doi: 10.1016/j.euf.2022.01.010, 35123928

[7] Grosso AA Salamone V Di Maida F Giudici S Cadenar A Lambertini L . Robot-assisted partial nephrectomy for renal cell carcinoma: a narrative review of different clinical scenarios. Asian J Urol. (2025) 12:210–6. doi: 10.1016/j.ajur.2024.09.010, 40458586 PMC12126952

[8] Gallo F Sforza S Mari A Luciani L Schenone M Minervini A. Robotic partial nephrectomy for bilateral renal masses. Curr Urol Rep. (2023) 24:157–63. doi: 10.1007/s11934-022-01143-4, 36538282

[9] Pandolfo SD Cerrato C Wu Z Franco A Del Giudice F Sciarra A . A systematic review of robot-assisted partial nephrectomy outcomes for advanced indications: large tumors (cT2-T3), solitary kidney, completely endophytic, hilar, recurrent, and multiple renal tumors. Asian J Urol. (2023) 10:390–406. doi: 10.1016/j.ajur.2023.06.001, 38024426 PMC10659988

[10] Gallo F Sforza S Luciani L Mattevi D Barzaghi P Mari A . Simultaneous robotic partial nephrectomy for bilateral renal masses. World J Urol. (2022) 40:1005–10. doi: 10.1007/s00345-021-03919-8, 34999905

[11] Carletti F Valenzi FM Tamborino F Turcan A Santarelli V Biasatti A . Predicting surgical outcomes in single-port robot-assisted partial nephrectomy: external validation and comparative analysis of PADUA, RENAL, and SPARE scores. World J Urol. (2025) 43:707. doi: 10.1007/s00345-025-06081-7, 41264016 PMC12634722

[12] Otoshi T Yamasaki T Hirayama Y Uchida J. Pilot experience of simultaneous robotic-assisted partial nephrectomy for bilateral renal tumors-single center analysis. Asian J Endosc Surg. (2021) 14:57–62. doi: 10.1111/ases.12831, 32602220

[13] Ko YH Ha JG Jang JY Kim YU. DaVinci SP-based simultaneous bilateral partial nephrectomy from the midline transperitoneal approach: a case report. J Yeungnam Med Sci. (2024) 41:1032. doi: 10.12701/jyms.2023.01032, 38196308 PMC10834266

[14] Wang H Cao D Moschovas MC Wu J Wang B Chang K . Early clinical experience with the Carina robotic platform in urologic surgery. BJUI Compass. (2025) 6:e70050. doi: 10.1002/bco2.70050, 40671868 PMC12266805

[15] Kim JK Lee H Oh JJ Lee S Hong SK Lee SE . Synchronous bilateral RCC is associated with poor recurrence-free survival compared with unilateral RCC: a single-center study with propensity score matching analysis. Clin Genitourin Cancer. (2019) 17:e570–80. doi: 10.1016/j.clgc.2019.02.008, 30922860

